# Phylogenetic, syntenic, and tissue expression analysis of *slc22* genes in zebrafish (*Danio rerio*)

**DOI:** 10.1186/s12864-016-2981-y

**Published:** 2016-08-12

**Authors:** Ivan Mihaljevic, Marta Popovic, Roko Zaja, Tvrtko Smital

**Affiliations:** 1 Division for Marine and Environmental Research, Laboratory for Molecular Ecotoxicology, Ruđer Bošković Institute, Bijenička cesta 54, 10000 Zagreb, Croatia; 2 Department of Oncology, University of Oxford, Old Road Campus Research Building, Roosevelt Drive, Oxford, UK; 3 Sir William Dunn School of Pathology, University of Oxford, Oxford, England UK

**Keywords:** SLC22, Zebrafish, Organic anion transporters, Organic cation transporters, Phylogeny, Synteny, Tissue expression

## Abstract

**Background:**

SLC22 protein family is a member of the SLC (Solute carriers) superfamily of polyspecific membrane transporters responsible for uptake of a wide range of organic anions and cations, including numerous endo- and xenobiotics. Due to the lack of knowledge on zebrafish Slc22 family, we performed initial characterization of these transporters using a detailed phylogenetic and conserved synteny analysis followed by the tissue specific expression profiling of *slc22* transcripts.

**Results:**

We identified 20 zebrafish *slc22* genes which are organized in the same functional subgroups as human *SLC22* members. Orthologies and syntenic relations between zebrafish and other vertebrates revealed consequences of the teleost-specific whole genome duplication as shown through one-to-many orthologies for certain zebrafish *slc22* genes. Tissue expression profiles of *slc22* transcripts were analyzed using qRT-PCR determinations in nine zebrafish tissues: liver, kidney, intestine, gills, brain, skeletal muscle, eye, heart, and gonads. Our analysis revealed high expression of *oct1* in kidney, especially in females, followed by *oat3* and *oat2c* in females, *oat2e* in males and *orctl4* in females. *oct1* was also dominant in male liver. *oat2d* showed the highest expression in intestine with less noticeable gender differences. All *slc22* genes showed low expression in gills, and moderate expression in heart and skeletal muscle. Dominant genes in brain were *oat1* in females and *oct1* in males, while the highest gender differences were determined in gonads, with dominant expression of almost all *slc22* genes in testes and the highest expression of *oat2a*.

**Conclusions:**

Our study offers the first insight into the orthology relationships, gene expression and potential role of Slc22 membrane transporters in zebrafish. Clear orthological relationships of zebrafish *slc22* and other vertebrate *slc22* genes were established. *slc22* members are mostly highly conserved, suggesting their physiological and toxicological importance. One-to-many orthologies and differences in tissue expression patterns of zebrafish *slc22* genes in comparison to human orthologs were observed. Our expression data point to partial similarity of zebrafish versus human Slc22 members, with possible compensatory roles of certain zebrafish transporters, whereas higher number of some orthologs implies potentially more diverse and specific roles of these proteins in zebrafish.

**Electronic supplementary material:**

The online version of this article (doi:10.1186/s12864-016-2981-y) contains supplementary material, which is available to authorized users.

## Background

Human SLC22 protein family belongs to the large Solute Carrier (SLC) superfamily of plasma membrane proteins which is a part of the Major Facilitator Superfamily (MFS) clan of proteins [1]. It is a highly diverse group of transport proteins comprising uniporters, symporters, and antiporters which facilitate the transport of a large number of substrates including ions, drugs, neurotransmitters, nucleosides, amino acids, and peptides across biological membranes. Furthermore, SLC22 transporters are important mediators of the ADME (absorption, distribution, metabolism and excretion) processes, thus maintaining homeostasis of various organic anions, cations and zwitterions, with an important role in toxicological response of the organism to numerous deleterious endo- and xenobiotics. Due to the described role, SLC22 transporters are dominantly expressed in barrier epithelia of liver, kidney and intestine, as well as in the brain and blood–brain barrier where they maintain homeostasis of organic ions [2].

SLC22 family consists of 13 functionally characterized transporters divided into several subfamilies. The classification is based on their specific functions, ranging from organic cation and anion transporters to organic zwitterion/cation transporters [3]. There are 23 *SLC22* genes in humans, whereas the overall number of identified *slc22* genes in other vertebrate species is variable and species-specific. The length of SLC22 proteins is 541 to 594 amino acids, with molecular masses ranging from 50 to 70 kDa, excluding posttranslational modifications. Most of them have twelve transmembrane α-helices which form the same number of transmembrane domains (TMD). The order and localization of TMDs structurally define the polyspecific active region of the SLC22 transporters [4].

There are three major groups of functionally characterized transporters within human SLC22 family. Their classification is based on functional properties and substrate specificities, and includes (1) organic cation transporters (OCTs, genes *SLC22A1-3*), (2) organic anion transporters (OATs, genes *SLC22A6-20*) and (3) organic cation/carnitine transporters (OCTNs, genes *SLC22A4-5*) [3]. Three members of human organic cation transporters (OCT1-3) are dominantly expressed in basolateral membranes of kidney and liver where they transport various organic cations. OCT substrates are type I organic cations (relative molecular mass below 400 gmol^−1^) and are variable in structure [5]. OCT/Oct co-orthologs broadly overlap in their substrate/inhibitor specificities. Typical human OCT substrates include model cations: 1-methyl-4-phenylpyridinium (MPP+), tetraethylammonium (TEA), 4-[4-(dimethylamino)-styryl]-N-methylpyridinium (ASP+), and 40,6-diamidino-2-phenylindol (DAPI) [4]; endogenous compounds such as acetylcholine, choline, putrescine, dopamine, epinephrine, norepinephrine, serotonin, histamine and agmatine [4, 6]; numerous xenobiotics such as metformin, acyclovir, berberine, cimetidine [3, 7, 8]; and toxins like aflatoxin B1 and ethidium bromide [9].

Organic cation/carnitine transporters (OCTNs) transport organic cations and zwitterions in a sodium-dependent and/or sodium-independent manner [4, 10], and are ubiquitously expressed in various tissues such as liver, kidney, ileum, colon [11], adipocytes, skeletal muscle cells [12] and blood–retinal barrier [4, 13]. Some of OCTN1 substrates are tetraethylammonium (TEA), acetylcholine [14], quinidine, pyrilamine, verapamil [15], and the anticancer drugs mitoxantrone and doxorubicin [4, 16]. OCTN2 is a major transporter of L-carnitine and plays a role in intestinal absorption and renal reabsorption of L-carnitine. Organic anion transporters (OAT) are ubiquitously expressed in almost all barrier epithelia throughout the organism. OAT1, 3, 5, 8 and 10 are dominant on the basolateral membranes, and OAT4 and URAT1 on apical membranes of renal proximal tubules where they excrete endogenous compounds such as prostaglandin E2 and F2, urate and acidic neurotransmitter metabolites [17, 18], medium chain fatty acids, α-ketoglutarate, citrulline, cyclic nucleotides [19, 20], and numerous small molecule xenobiotics [21]. OAT2, 7 and 9 are mainly expressed on basolateral membranes of hepatocytes [22–24] where they mediate hepatic excretion of endogenous substrates such as estrone sulfate, dehydroepiandrosterone sulfate and butyrate, as well as antiviral drugs and ochratoxin A [25].

However, despite their polyspecificity and physiological and/or toxicological significance well-recognized in mammals, the knowledge on Slc22 transporters in non-mammalian species is truly modest. Among invertebrate Slc22 transporters, Octs have been identified on the genome level in fruit fly (*Drosophila melanogaster*) and *Caenorhabditis elegans* [26]. In vertebrates, apart from being identified on genomic level trough zebrafish genome project [27], there has been a limited number of studies on Slc22 transporters, or uptake proteins in general. Several studies on zebrafish Slc21 family were reported by our group, with a comprehensive identification and phylogenetic and expressional characterization [28] followed by more detailed functional characterizations of zebrafish Oatp1d1 [29, 30]. Members from the Oat subfamily in winter flounder (*Pseudopleuronectes americanus*) have been partly characterized by Ashlamkan et al. [31] and Wolff et al. [32]. They also reported one *Oat* gene sequence in zebrafish and one gene in pufferfish (*Takifugu rubripes*) that are similar to the winter flounder *Oat*. Additionally, Smith et al. [33] and Chatsudthipong and Dantzler [34] partially characterized sodium-coupled transport of organic anions in *Cancer borealis* and garter snake (*Thamnophis spp*.).

Therefore, due to evident lack of knowledge on Slc22 transporters in non-mammalian species, in this study we performed the first comprehensive identification and elucidation of orthological relationship among SLC22/Slc22 proteins of fish and other higher vertebrates. In order to do so we have used zebrafish (*Danio rerio*) as a highly important vertebrate model species in biomedical and environmental research. Our approach was based on detailed phylogenetic and conserved synteny analysis, followed by tissue specific expression profiling of Slc22 transcripts. In addition, the obtained data allowed the first insight into possible roles of these membrane transporters in zebrafish.

## Methods

### Phylogenetic analysis

Designation of gene and protein names used throughout the text is based on the Zebrafish Nomenclature Guidelines: (https://wiki.zfin.org/display/general/ZFIN+Zebrafish+Nomenclature+Guidelines); e.g., fish: shh/Shh, human: SHH/SHH. Gene and protein sequences were retrieved from NCBI [35] and ENSEMBL [36] databases using blastx algorithm. Following species were included in the phylogenetic analysis: mammals – human (*Homo sapiens*) and mouse (*Mus musculus*); bird – chicken (*Gallus gallus*); reptile – anole lizard (*Anolis carolinensis*); amphibian – frog (*Xenopus laevis*); actinopterygian or ray-finned fishes – zebrafish (*Danio rerio*), pufferfishes Japanese pufferfish (*Takifugu rubripes*) and green spotted pufferfish (*Tetraodon nigroviridis*), Atlantic cod (*Gadus morhua*), stickleback (*Gasterosteus aculeatus*), medaka (*Oryzias latipes*) and rainbow trout (*Oncorhynchus mykiss*); sarcopterygian or lobe-finned fish – West Indian Ocean coelacanth (*Latimeria chalumnae*); and a tunicate – sea squirt (*Ciona intestinalis*). MUSCLE algorithm [37] was used for alignment of *SLC22/slc22* sequences and phylogenetic tree was constructed using Maximum Likelihood method in PhyML 3.0.1 software [38]. Confidence of nodes was estimated by approximate likelihood ratio test (aLRT) [39]. Based on the phylogenetic relationships, all previously unclassified genes were provisionally annotated. Names were given in accordance with the new nomenclature adopted by the HUGO Gene Nomenclature Committee [40].

Orthology predictions using syntenic relationships between zebrafish and human genes of interest were made using Genomicus [41], a conserved synteny browser synchronized with genomes from the Ensembl database [42].

Analysis of transcription factor binding motifs was made using AliBaba 2.1, a web-based server for prediction of transcription factor binding sites by constructing matrices on the fly from TRANSFAC 4.0 sites. Analysis was conducted using default parameters. Amino acid sequence motif comparison was performed using the Multiple Expectation-maximum for Motif Elicitation (MEME) suite (http://alternate.meme-suite.org/tools/meme) [43]. Detection of motifs was performed for 41 selected zebrafish and human Slc22/SLC22 sequences with a threshold of 16 motifs and amino acid length of 6–10 using normal discovery mode.

### Tissue-specific gene expression analysis: RNA isolation, reverse transcription and qRT-PCR

Adult, approximately nine month old female and male zebrafish of the AB strain were purchased from a certified local supplier. All zebrafish specimens that were sacrificed for tissue/RNA isolations were anesthetized with overdose of tricaine methane sulfonate (MS222, 200 mg/L) via prolonged immersion, followed by one of the established confirmatory methods [44]. In order to collect sufficient amount of kidney tissue for RNA extraction we pooled kidneys form 15 individual fish. The rest of isolated tissues (brain, gills, liver, intestine, heart, skeletal muscle, eye and gonads) were pooled in three independent pools of five individual fish. Tissues were stored in RNA later (Qiagen, Hilden, Germany), and afterwards homogenized for 20 s using rotor-stator homogenizer (Ultra-turrax T25, IKA, Germany) at 10,000 rpm. Total RNA isolation was carried out using Rneasy Mini Kit (Qiagen, Hilden, Germany). For the purpose of RNA extraction we used 20 mg of each tissue. Genomic DNA digestion was carried out using Rnase-free DNase Set (Qiagen, Hilden, Germany). RNA was quantified, and 260/280 and 260/230 nm ratios were analyzed using BioSpec nano micro-volume spectrophotometer (Shimadzu, Kyoto, Japan). RNA integrity was checked visually by gel electrophoresis. cDNA was produced from 1 μg of total RNA using High Capacity cDNA Reverse Transcription Kit with Rnase Inhibitor (Applied Biosystems, Foster City, CA, USA).

For the purpose of quantitative Real time PCR (qRT-PCR), specific primers were designed in the Primer express 3.0 Software (Applied Biosystems, CA, USA), adjusted manually if necessary and purchased from Invitrogen (Carlsbad, CA, USA) (Table 1). Target amplicons of 90–100 bp were cloned using pGEM-T Vector System I (Promega, Madison, WI, USA). Plasmids were purified by QIAprep Spin Miniprep Kit (Qiagen, Hilden, Germany) and amplicons were verified by sequencing at the Rudjer Boskovic Institute DNA Service (Zagreb, Croatia). Primer efficiencies were determined using the recombinant pGEM-T as a template for each primer pair. Primer concentrations were optimized combining three primer concentrations: 300, 600 and 900 nM. Primer concentrations resulting in the highest fluorescence signal at the lowest Ct number were chosen as optimal. Primer sequences, optimal concentrations and primer efficiency of target gene sequences are given in Table 1, while accession numbers of target genes are given in the Additional file 1: Table S1. For the purposes of the inter-gene comparison throughout one tissue, relative quantification was used as method of choice. Target genes were normalized to the housekeeping gene (HKG) using Q-Gene application (http://www.qgene.org/) for the processing of qRT-PCR data (described in detail by Muller et al. [45] and Simon [46], according to the equation (1):

**Table 1:** Primers used in the quantitative Real time PCR (qRT-PCR)

Protein name	Primer sequence 5′ -> 3′	T_a_	Final conc. (nM)	Efficiency (%)
Oatl	F TGCTGTTCTGATCTTGGACGA	62	300	92
	R TGCT ATTAAACCAGCGAT GAC	60	300	
Oat3	F GGGTCAGCATTTACCTCATCCA	60	300	105
	R GATGGCCGTCGTCCTAACAT	58	300	
Oat2a	F TCGCCATTGCAAGAACCTTAT	58	300	92
	R AAGGTGCGATGCTTAACATCTG	58	300	
Oat2b	F GATTGTAAGTGTTCCAGCACAAGAA	58	300	101
	R TGAGCTGCTGGACGAGTTTATC	58	300	
Oat2c	F GCACTTTGATAACAGCACCTTCAT	58	300	95
	R GAAGAAGATGGTGGTTGTCAATTTC	59	300	
Oat2d	F ACAGTATGGCATGGGCTGTT	60	300	100
	R AAGGTGAAGTGACAGCCACT	60	300	
Oat2e	F GGTGTTATGAtCAGTTTGGATT	60	300	95
	R TTGGAGCAGTTACTGTGAGG	58	300	
Octl	F GAGTCACAGGGATTCTGGT	58	300	99
	R ACCATCCAACCGCCCTTCA	60	300	
Oct2	F TGGCCTTGGAGTCTCTGG	60	300	99
	R TGGCGGTCCATGCTCCTTT	60	300	
Octnl	F GCTCTGGAATCGGGCAGAT	60	300	100
	R GGCCAGTGAGGGCGTTTG	60	300	
Octn2	F CACCGCCTCACTGGCCAACC	60	300	112
	R ATCTCTTCCTGGAAAGCTT	60	300	
Oct6	F GCTGTAGGGAGTGGTAGTAT	60	300	96
	R CGATGAGCTGCGGCAGATA	60	900	
Orctl3	F ACCTCGTGTCCCAGTTCATT	60	300	100
	R TGCATATGATCTCTGGGCGA	60	300	
Orctl4	F TCACCGCCTTCCACATGTT	59	300	96
	R TTGGAGCCTGTCGGAGGAT	59	300	
Eflα	F CCTGGGAGTGAAACAGCTGATC	60	300	96
	R GCTGACTTCCTTGGTGATTTCC	60	300	

1 $$ \mathrm{M}\mathrm{N}\mathrm{E} = \left(\left({\mathrm{E}}_{\mathrm{ref}}\right)\ \hat{\mkern6mu} \mathrm{C}{\mathrm{t}}_{\mathrm{ref},\mathrm{mean}}\right)\ /\ \left(\left({\mathrm{E}}_{\mathrm{t}\mathrm{arget}}\right)\hat{\mkern6mu} \mathrm{C}{\mathrm{t}}_{\mathrm{t}\mathrm{arget},\ \mathrm{mean}}\right) $$

where MNE stands for mean normalized expression; E_ref_ is housekeeping gene efficiency; E_target_ is target gene efficiency; Ct_ref_, _mean_ is mean Ct value for the housekeeping gene; and Ct_target, mean_ stands for mean Ct value of the target gene. Data are presented as gene of interest expression relative to the housekeeping gene expression multiplied by the factor of 10,000. Elongation factor (EF1α) was chosen as a housekeeping gene given the fact that its expression was similar across all analyzed tissues. Expression was considered to be very high for MNE > 8000*10^5^ (C_t_ < 19), high for MNE 800*10^5^ - 8000*10^5^ (C_t_ 20–23), moderate for MNE 30*10^5^ - 800*10^5^ (C_t_ = 23–26), and low for MNE < 30*10^5^ (C_t_ >27).

qRT-PCR was performed using the ABI PRISM 7000 Sequence Detection System using Power SYBR Green PCR Master Mix (Applied Biosystems, Foster City, CA, USA). The reaction mix was prepared to a final volume of 10 μl containing: 5 μl of SYBER Green master mix, 0.5 μl of each primer (of adequate concentration), 1 μl of template (10 ng/well) and 3 μl of Ultrapure Dnase/Rnase free distilled water (Molecular Bioproducts, San Diego, CA, USA). After the denaturation at 95 °C for 10 min, 40 cycles of amplification were carried out with denaturation at 95 °C for 15 s, annealing and elongation at 60 °C for 1 min altogether followed by the melting curve analysis. Data were analyzed with ABI PRISM Sequence Detection Software 1.4 (Applied Biosystems, Foster City, CA, USA) and GraphPad Prism Software version 5.00.

## Results

### Gene identification and phylogenetic analysis

**Fig. 1:**
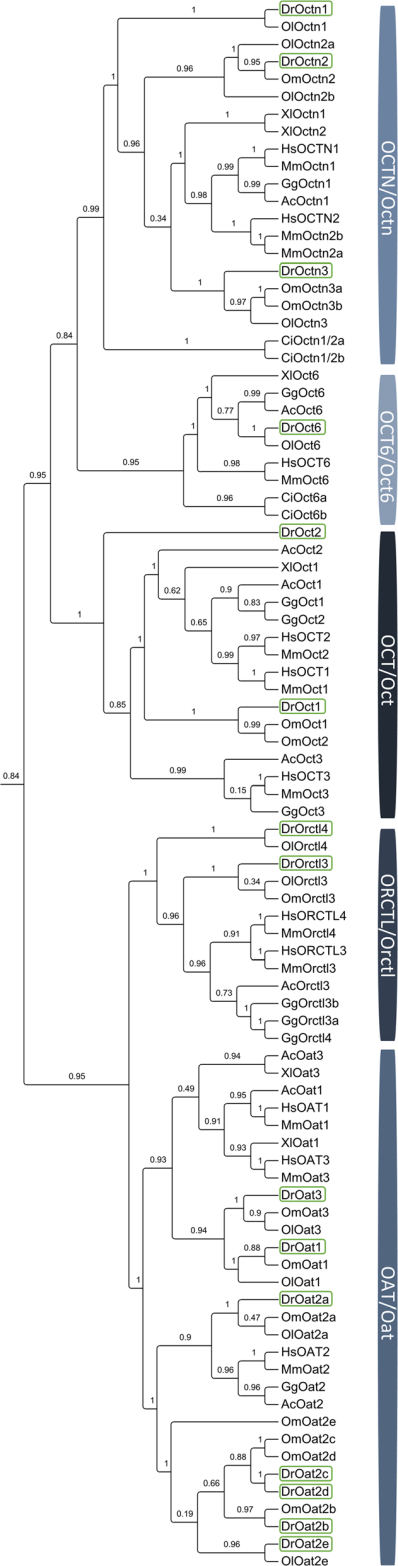
Phylogenetic tree of SLC22/Slc22 protein family in vertebrates. Species abbreviations: Hs, *Homo sapiens*; Gg, *Gallus gallus*; Ac, *Anolis carolinensis*; Xl, *Xenopus leavis*; Dr, *Danio rerio*; Om, *Oncorhynchus mykiss*. Zebrafish genes are framed in green

Detailed identification of *slc22* genes in representative vertebrate species and *C. intestinalis* have revealed total of 20 zebrafish *slc22* genes (Fig. 1, Additional file 1: Figure S1, Additional file 2). Two of them, *slc22a1* and *slc22a2*, belong to the subgroup of organic cation transporters (*OCTs*). In higher vertebrates (reptiles to humans), three *OCT/Oct* orthologs are present, while in lower vertebrates (i.e., fish and amphibians) there are two *Oct* orthologs. The only exception is amphibian *X. laevis* with one *Oct* ortholog (Fig. 1). Phylogenetic tree revealed clear clustering of higher vertebrates *OCTs/Octs* separately from fish orthologs, with the exception of zebrafish *Oct2* and coelacanth *Oct3a* and *Oct3b* (Fig. 1). Amino acid sequence identities within vertebrate OCT/Oct subfamilies are 42–53 %, whereas among fish Oct proteins identities are 43–72 %.

*OCTN/Octn* group is well conserved from fish to mammals with two members in each tetrapod species: *OCTN1/Octn1* and *OCTN2/Octn2*. Two co-orthologs of these human genes are found in all vertebrates including zebrafish. Additionally, *slc22a21* gene subcluster is positioned within the *Octn* cluster, indicating that these genes might be novel orthologs of *Octn* subfamily (Fig. 1). OCTN/Octn subfamily shares 44–52 % amino acid sequence identity within vertebrates, whereas OCT6/Oct6 orthologs share 39–59 %, and 70–74 % identity among vertebrates and among teleost species, respectively.

Within the subgroup of organic anion transporters (*OATs*), 7 genes were found. Within the *OAT1/OAT3* cluster, two genes are present in each tetrapod species, as well as in each analyzed teleost species. However, teleost subcluster is distinct from the tetrapod cluster and direct orthologs of human *OAT1* and *OAT3* in fish species cannot be distinguished among each other. That is the reason why all teleost transporters are provisionally annotated as Oat1 and/or Oat3 in the order of appearance within the phylogenetic tree (Fig. 1). Phylogenetic analysis of *OAT2/Oat2* showed one-to-many orthology between human and zebrafish genes, revealing five zebrafish Oat2 (a-e) co-orthologs (Fig. 1). The third cluster of *OAT*s includes *OAT4-7* and *URAT1* genes. These genes are found only in mammals and do not have orthologs in other vertebrates (Fig. 1). Relatively high amino acid sequence identity was revealed between vertebrate OAT1/Oat1 and OAT3/Oat3 subfamily (46-56 %) which is the same identity percentage as within each separate subfamily. Oppositely, OAT2/Oat2 subfamily showed lower amino acid identity with OAT1/OAT3 cluster (28-30 %), with 40-54 % identity within vertebrate OAT2/Oat2 subfamily.

*SLC22A13*, also known as organic cation transporter-like 3 (ORCTL3) or OAT10, and *SLC22A14*, known as organic cation transporter-like 4 (ORCTL4), together with their co-orthologs in other vertebrates form a distinct cluster that is closer to the OAT than to OCT or OCTN group (Fig. 1).

Within human *SLC22* family, there are several subfamilies of orphan genes which are also present in zebrafish, namely *slc22a15* (FLIPT1), *slc22a17* (BOIT), *slc22a23* and *slc22a31*, whose functions are still unknown. Phylogenetic analysis revealed their specific clustering and clear one-to-one orthology relationships to corresponding genes in other vertebrate species (Fig. 1).

Accession codes and annotations of protein sequences for all chordate SLC22/Slc22 family members are given in the Additional file 1 (Table S1).

### Conserved synteny analysis

**Fig. 2:**
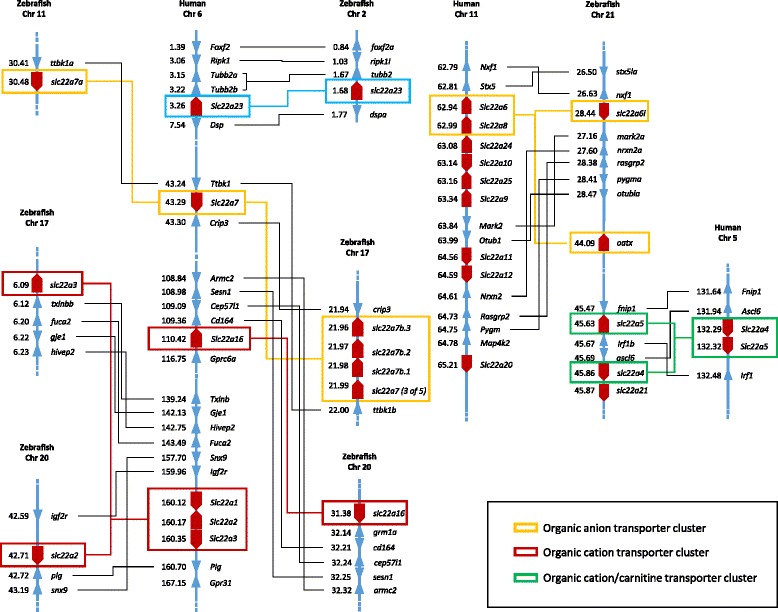
Conserved synteny analysis of human and zebrafish *SLC22*/*slc22* genes. Numbers next to the gene names represent megabase pair (Mbp) of particular gene location on the chromosome

Human *OCT* (*SLC22A1-3*) genes are located within one cluster on chromosome 6, between 160.12 and 160.35 megabase pair (Mbp), with forward orientation of *OCT1* and *OCT3* and reverse orientation of *OCT2* (Fig. 2). Interestingly, there are other *SLC22* members present on human chromosome 6, such as *OAT2*, *OCT6* and *SLC22A23*. Syntenic comparison revealed conserved synteny of zebrafish *oct1* (chromosome 20) with human *OCT* cluster (Fig. 2). Neighboring genes of zebrafish *oct1* matched the immediate gene environment of the human ortholog cluster. *igf2r* gene on zebrafish chromosome 20 is located upstream at 42.59 Mbp, next to *oct1* gene, in the same forward orientation as human *IGF2R. plg* and *snx9* genes, which are located downstream of *oct1*, showed opposite reverse orientation in comparison with human orthologs, with *snx9* located downstream of *oct1*, whereas the human ortholog is located upstream of *OCT* cluster (Fig. 2). Another zebrafish ortholog, *oct2*, is located on chromosome 17. Synteny analysis of *oct2* showed conserved syntenic relationship with human *OCT* cluster which was confirmed by analysis of four neighboring genes, *txlnbb*, *fuca2*, *gje1* and *hivep2*, all located downstream of *oct2* with the same orientations as human orthologs (Fig. 2).

Phylogenetic and synteny analysis revealed five zebrafish *oat2* co-orthologs, separated on two chromosomes (chr 11 and chr 17) (Fig. 2). Zebrafish *oat2a* (*slc22a7a*) is positioned on chromosome 11 with only one neighboring gene *ttbk1a* in the same arrangement and orientation as human ortholog OAT2 (*SLC22A7*) (Fig. 2). The other four zebrafish orthologs (*oat2b-e, slc22atb.3, slc22a7b.2, slc22a7b.1, slc22a7 (3 of 5)*) are located in a cluster on chromosome 17. Analysis revealed syntenic relationship between zebrafish *oat2b-e* cluster and human *OAT2* based on two neighboring genes, *crip3* and *ttbk1b*, located on opposite sides and with opposite orientation than human orthologs (Fig. 2).

Another *SLC22* gene on human chromosome 6 is *OCT6* (*SLC22A16*) at 110.42 Mbp, located between *OAT2* and *OCT* cluster (Fig. 2). Human *OCT6* showed syntenic relationship with single zebrafish ortholog on chromosome 20 at 31.38 Mbp as was determined based on five orthologous neighboring genes. Finally, the last of *SLC22* genes on human chromosome 6 is *SLC22A23* in the beginning of the chromosome, at 3.26 Mbp, which is syntenic to zebrafish *slc22a23* as was based on four neighboring genes (Fig. 2).

Most of human *SLC22* genes are located on chromosome 11: nine *SLC22* genes are organized in one big cluster of six genes (*SLC22A6, 8, 9, 19, 24* and *25*), one small cluster of two genes (*SLC22A11* and *12*) and one isolated gene (*SLC22A20*) (Fig. 2). Majority of these genes are specific for mammals and there are no orthologs in zebrafish. Only two genes showed phylogenetic and syntenic relationship with zebrafish orthologs, *SLC22A6* and *8*. They showed syntenic relationship with zebrafish *oat1* (*oatx*) and *oat3* (*slc22a6l*) on chromosome 21 which was confirmed with seven neighboring genes (Fig. 2). However, although neighboring genes remained close to *oat1* gene their distribution and orientation drastically changed in comparison to human orthologs. Interestingly, zebrafish *oat3*, localized far form *oat1* at 44.09 Mbp, showed no syntenic relationship with human ortholog *OAT3* (*SLC22A8*) (Fig. 2). There are three more zebrafish *slc22* genes on chromosome 21 close to *oat3*: *octn1* (*slc22a4*), *octn2* (*slc22a5*) and *slc22a21. octn* genes showed syntenic relationship with human orthologs OCTN1 and OCTN2 on chromosome 5 (confirmed with three neighboring genes) (Fig. 2).

Two representatives of human *ORCTL* subfamilies, *SLC22A13* and *SLC22A14*, are located in a cluster on chromosome 3 and are syntenic to zebrafish orthologs on chromosomes 24 and 17, respectively (Figure S2). There is one more *slc22* gene on zebrafish chromosome 17, *slc22a15* at 19.48 Mbp. However, it did not show any conserved genetic neighborhood with human ortholog on chromosome 1 (Figure S2). Zebrafish gene *slc22a17* on chromosome 24 at 41.09 Mbp showed weak syntenic relationship with human ortholog on chromosome 14, which was confirmed with only one neighboring gene, *MYH6* (Figure S2).

Finally, zebrafish genes *slc22a18* and *slc22a31* are both located on chromosome 7 at 39.27 and 56.13 Mbp, respectively. They showed syntenic relationship with human orthologs, with neighboring genes that remained in the same orientation (Additional file 1: Figure S2).

### Gene regulation and specific sequence motif analysis

*In silico* prediction of transcription factor binding sites revealed several motifs for hepatocyte nuclear factor 1, 3 and 4α (HNF-1, 3, 4α), activating transcription factor 1 (ATF1) and cAMP responsive element binding protein 1 (CREB1) (Additional file 3). Analysis also revealed presence of motifs for steroid hormone receptors such as corticoid receptors (GR), progesterone receptor (PR), androgen receptor (AR), estrogen receptor (ER) and thyroid hormone receptor (T3R).

Multiple Expectation-maximum for Motif Elicitation (MEME) revealed 16 Slc22/SLC22 specific motifs (Additional file 4). All zebrafish and human Slc22/SLC22 transporters have 13 amino acid long, major facilitator superfamily signature (MFS), G(RKPATY)L(GAS)(DN)(RK)(FY)GR(RK)(RKP)(LIVGST)(LIM) between the second and the third transmembrane domain (TMD), together with conserved amphiphilic solute facilitator (ASF) domain located before TMD2. Analysis also revealed 14 amino acid long Oat/OAT – specific motif in the large loop between TMD1 and 2 (Additional files 4 and 5).

### Tissue expression profiles

**Fig. 3:**
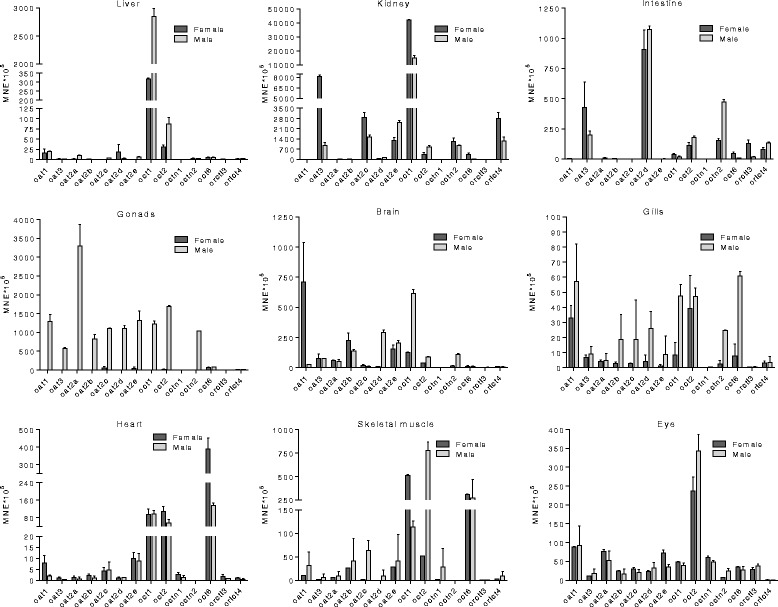
Tissue expression profile of major zebrafish *slc22* genes. Results of three independent measurements (three pools) are given, except in the case of kidney (one pool of 15 individuals). Tissue expression results are presented as mean values ± SEM from 3 to 5 pools. MNE stands for mean normalized expression normalized to the housekeeping gene elongation factor 1α (EF1α)

In zebrafish liver we observed high expression of *oct1* transcript in males, followed by moderate expression *oct1* in females and *oct2* in both sexes (Fig. 3, Additional file 1: Table S2). More diverse *slc22* expression pattern was found in kidney. Very high expression was observed for *oct1* in both sexes and *oat3* in females. *oat2c*, *oat2e*, *octn2* and *ortcl4* showed high expression in both sexes, and *oct2* in females. Moderate expression was observed for *oct2* in males, *oct6* in females and *oat2d* in both sexes (Fig. 3). Most pronounced gender differences in *slc22* expression in kidney were observed for *oct6* and *oat3* which are 22- and 8-fold more expressed in females, respectively (Fig. 3).

In the intestine, the levels of expression of *slc22* genes are not as high as in liver and kidney (Fig. 3, Additional file 1: Table S2). Dominant gene found in intestine are *oat2d*, present at high expression levels in both sexes, followed by moderate expression of *oat3*, *octn2*, *oct2*, and *orctl4* in both sexes. Most pronounced gender expression difference was observed for *orctl3* that is 7 times higher expressed in females.

In zebrafish gills, *slc22* genes are generally lower expressed in comparison to other tissues (Fig. 3, Additional file 1: Table S2). *oct6* showed moderate expression in males, followed by *oct1*, whereas *oat1* and *oct2* showed similar moderate expression in both genders (Fig. 3). In the brain, most of investigated genes showed moderate expressions with high expression difference among genders observed for *oat1*, which is 30 times higher expressed in females, as well as for *oat2d* and *oct1* which showed 25 times higher expression in males (Fig. 3). In zebrafish heart, organic cation transporters (*oct1, 2* and *6*) showed expression in moderate range, whereas other *slc22* genes showed low expression (Fig. 3, Additional file 1: Table S2). Similar expression pattern with dominant expression of organic cation transporters in moderate range was shown in skeletal muscle (Fig. 3, Additional file 1: Table S2). In zebrafish eye, *oct2* is moderately expressed, followed by *oat1*, *oat2a* and *octn1* (Fig. 3, Additional file 1: Table S2).

In the gonads, expression pattern *slc22* genes differs the most among genders (Fig. 3, Additional file 1: Table S2). In testes, most of the genes show moderate to high expression, with only three low expressed genes: *octn1*, *orctl3* and *4* (Fig. 3). The dominant *slc22* gene in testes is *oat2a* (high expression), followed by high expression of *oat1*, *oat2b-e*, *oct1*, *oct2* and *octn2* and moderate expression of *oat3* and *oct6* (Fig. 3). On the other hand, only *oat2c*, *oat2e* and *oct6* showed moderate expression levels in ovaries, whereas low expression was determined for all the other genes (Fig. 3).

## Discussion

Previous research on human and other mammalian SLC22/Slc22 proteins showed the presence of 23 human *SLC22* genes out of which 13 were functionally characterized [47]. Data obtained in this study offer the first insight into the zebrafish Slc22 membrane transporters, and our phylogenetic and synteny analysis revealed orthological relationships of fish Slc22 with related transporters in other vertebrate species.

Two identified members of zebrafish organic cation transporters (Octs) showed three-to-two gene orthology comparing with human OCTs (Fig. 1). All vertebrates, starting from fish to amphibians, have two *oct* members, whereas reptiles and other higher vertebrates have three *OCT*/*Oct* genes (Fig. 1). Our synteny analysis allowed better insight into the orthological relationship between human and zebrafish *OCTs/octs*, revealing that human *OCT* cluster located on chromosome 6 shows clear syntenic relationship with zebrafish *oct* genes separated on chromosomes 17 and 20 (Fig. 2). This separation of usually clustered *slc22* genes may suggest different evolutionary pathways which could have lead towards different functional roles of zebrafish Octs [48, 49]. That possibility is additionally emphasized with significantly different tissue expression profiles of *oct1* and *oct2*, especially in comparison with human *OCT*s [9]. Very high expression of *oct1* in kidney followed by high expression in liver and moderate expression in brain is similar expression pattern to the human *OCT1* and *2.* It points to the possibility that Oct1 in zebrafish could play similar roles as human OCT1 and *2* [50]. On the contrary, moderate expression of *oct2* throughout all examined tissues, apart from testes and kidney, suggests a more specific physiological role and possible involvement in the elimination of endogenous cations and drugs through kidneys (Fig. 3). Accordingly, zebrafish *oct2* expression pattern is different than human *OCT2*, which is primarily expressed in kidney, followed by brain, intestine, placenta, lungs and inner ear [51, 52]. Furthermore, considering that zebrafish do not have an *OCT3* ortholog, it is possible that Oct1 and 2 transporters in zebrafish compensate for the function of Oct3, especially considering extensive substrate overlap among mammalian OCT1, 2 and 3 [4, 51, 53]. This assumption is additionally supported by the highest expression of *oct2* in comparison to other examined *slc22* transcripts in zebrafish eye, which is similar to expression of human and murine OCT3/Oct3 in the eye, where it plays an important role in absorption, distribution and clearance of various xenobiotic substrates [54, 55]. Organic cation transporters also showed gender dependent differences in tissue expression. The observed difference might be consequence of differential gene regulation with steroid hormones which can occur not only in gonads but also in other tissues [56]. Identified steroid – dependent regulatory elements in the *slc22* promoter regions also support the observed expressional differences.

OCTN/Octn transporters have an important physiological role in vertebrates, primarily in the transport of L-carnitine, an essential compound in the fatty acid metabolism [57]. We have found that OCTN/Octn transporters are highly evolutionary conserved and are present in all analyzed vertebrate species, ranging from primitive chordate *Ciona intestinalis* to human (Fig. 1). Apart from OCTN1 and 2 orthologs, we have identified the third *octn* gene in zebrafish, *slc22a21*, which is present only in fish species (Fig. 1). The expression pattern of zebrafish *octn1* and *octn2* differs in comparison to their human orthologs. Zebrafish *octn1* showed only moderate expression in zebrafish eye, and its expression in other examined tissues was low, whereas human *OCTN1* is ubiquitously expressed including kidney, intestine, brain, testes, lung and heart [11, 50, 58–60]. Zebrafish *octn2* is highly expressed in kidney and testes and moderately expressed in intestine, a pattern that in part corresponds to the expression of human and rodent *OCTN2*/*Octn2. OCTN2*/*Octn2* is expressed in the kidney and intestine but not in the testes of mammals [3, 11]. Taking into account high renal expression of zebrafish *octn2*, we hypothesize it might be responsible for the secretion and reabsorption of organic cations in zebrafish kidney, similar to its mammalian ortholog OCTN2 [15]. Absence of *octn* transcript in zebrafish heart and skeletal muscle could be explained by higher expression of *oct6* in these tissues which indicate that Oct6 transporter could play compensatory role in carnitine transport.

Similar to *octn*, we found that *OCT6*/*oct6* is highly conserved *SLC22*/*slc22* gene within the vertebrate phyla. It is present in all investigated vertebrates from *C. intestinalis* to human, with clear one-to-one orthology. (Fig. 1). However, there is a partial divergence in tissue expression pattern in comparison to human OCT6. Human OCT6 is primarily expressed in testes, where it may be involved in regulation of L-carnitine and spermidine concentrations in spermatozoa [3]. Human and rodent *OCT6*/*Oct6* is also present in kidney, liver, brain, heart and other organs, although its function in these organs is still unknown [61–64]. Zebrafish *oct6* showed moderate expression in fibrous tissues like skeletal muscle and heart, and is moderately expressed in female kidney, gonads, male gills and female intestine (Fig. 3). Its moderate expression in testes and clear orthology relationship to *OCT6* may suggest its potential role in transport of L-carnitine and spermidine in testes, while the role in other organs remains to be addressed in future studies.

We have identified seven organic anion transporter genes in zebrafish. One-to-one orthology of *oat1* and *oat3* to human *OAT1* and *OAT3* (Fig. 1) points to high degree of conservation of OAT1 and OAT3 function in the physiology of vertebrates. However, tissue expression data partially differ among zebrafish and human orthologs, especially in the case of *oat1*. Zebrafish *oat1* is primarily expressed in testes and female brain (high expression), while human *OAT1* and mouse *Oat1* are primarily expressed in kidney and brain [65]. Zebrafish *oat3* showed more ubiquitous tissue expression than *oat1*, with dominance in kidney, especially in females, followed by moderate expression in testes, intestine and brain. In human and mouse *OAT3*/*Oat3* is present in kidney and liver where it is responsible for transport of various xenobiotics [21, 65]. Considering high expression of zebrafish *oat3* in kidney, it could have partially overlapping function with its human ortholog OAT3 in the transport and subsequent elimination of xenobiotics. Potential functional similarities of zebrafish and human organic anion transporters are also supported by the identified Oat/OAT – specific amino acid sequence motif, long with more conserved MFS and ASF domains (Additional file 4). These evolutionary conserved regions could be the structural basis of the Slc22 family, whereas class – specific motif may have functional features, allowing the flexibility for polyspecific substrate transport [66].

Five members of *oat2* (*a - e*) subfamily were identified in zebrafish (Fig. 1). Interestingly, *OAT2/oat2* genes of higher tetrapods showed one-to-one orthologies, whereas all investigated fish species showed one-to-many orthologies with human genes. This may be a consequence of the independent whole genome duplication (WGD) in teleost fish, an additional WGD in salmonids (e.g., rainbow trout) and additional individual gene or gene cluster duplications [27, 67]. The presence of only one *OAT2*/*oat2* ortholog in all other examined tetrapods suggests that the second round of genome duplication might have been a trigger for diversification of *oat2* genes [68]. Our conserved synteny analysis confirmed multiple gene duplication of zebrafish genes. It showed double conserved synteny of five zebrafish *oat2* genes on chromosomes 11 (*oat2a*) and 17 (*oat2b-e*) with human *OAT2* on chromosome 6 (Fig. 2). None of the zebrafish *oat2* co-orthologs corresponds to the human *OAT2* in terms of expression profiles. Human *OAT2* is dominant in liver, moderately expressed in kidney, and low expressed in testes, intestine and uterus [22], whereas none of the zebrafish *oat2* genes is dominant in liver (Fig. 3). Similarity in expression profiles is found for *oat2c* and *o*at2e which are highly expressed in kidney (Fig. 3), like human OAT2 [18, 22]. Unlike human and rodent *OAT2/Oat2*, all zebrafish *Oat2* members (*Oat2a - e*) were highly expressed in testes and moderately expressed in brain. The divergence in terms of orthology relationships and expression profiles point to potentially different roles of zebrafish Oat2 transporters in comparison to mammalian OAT2/Oat2. The only overlap is present in respect to the renal expression of *oat2c* and *oat2e*, suggesting their functional similarities with human OAT2, which plays important roles in renal-handling of creatinine, secretion of uric acid and other numerous xenobiotics [64, 69].

Human ORCTL4 transporter has been identified only on the genome level, without known function, whereas ORCTL3 (OAT10) [3] was found to be responsible for the uptake of vitamin B_3_ (nicotinate) in the intestine and urate in the kidney [70]. Our results on tissue distribution of zebrafish *ortcl3* correspond to the human *ORCTL3* expression (high expression in kidneys, moderate in intestine) (Fig. 3), which would suggest similar physiological role of ORCTL3/Ortcl3 from zebrafish to mammals. Considering clear one-to-one orthologies of OAT10 within the whole vertebrate subphylum, as well as overlapping expression profiles between zebrafish and human Oat10/OAT10 (high expression in kidneys, moderate in intestine) (Fig. 3), we suggest a conserved role of Oat10.

## Conclusions

Data from this study provided new insights into orthology relationships of SLC22/Slc22 transporters among fish and other vertebrates, and offered the first integral evidence on Slc22 transporters in zebrafish as a highly relevant vertebrate model species. Phylogenetic and synteny analysis of *SLC22*/*slc22* genes point to certain similarities among human (and mammalian in general) and zebrafish Slc22 transporters. Moreover, clear one-to-one orthology, conserved synteny and gene conservation within the whole vertebrate subphylum imply physiologically important roles for the majority of Slc22 transporters. Those similarities can be most directly observed within the Octn group, indicating possible important role of zebrafish Octn2 in maintaining renal organic cation homeostasis. Octn2 may be involved in transport of L-carnitine as a major physiological substrate, Oct6 could have evolutionary conserved physiological function in transport of L-carnitine and spermidine, and finally, due to its dominant expression in liver and kidney zebrafish Oct1 may have similar function as human OCT1 and OCT2. Other zebrafish Slc22 members showed less conserved similarities with human and other vertebrate SLC22/Slc22 transporters, possibly due to the gene divergence following the teleost specific WGD. This specific evolutionary event resulted in emergence of novel *slc22* genes in zebrafish, which were changing, multiplying or disappearing during the course of evolution. This in turn resulted in higher diversity of *slc22* genes which is especially evident in the group of organic anion transporters. Consequently, due to occurrence of novel functional changes during the evolution of zebrafish *slc22* genes their expression profiles are partly different in comparison to human orthologs. However, taking into account the described high expression of certain *slc22* genes (e.g., *oct1*, *oat3*, *oat2c*, *oat2d*, *oat2e*, *octn2* and *orctl4*) in toxicologically important organs/tissues, as well as polyspecific substrate preferences of their human orthologs, we hypothesize that these transporters probably have important roles in defense against various deleterious endo- and xenobiotic substances, making the Slc22 transporters crucial elements and determinants in ADME/Tox processes in zebrafish. Based on data from this study, functional role(s) of zebrafish Slc22 transporters remains to be addressed in further studies directed to detailed functional characterization of single transporters in suitable expression systems.

## Abbreviations

ADME, absorption, distribution, metabolism and excretion; ASP+, 4-[4-(dimethylamino)-styryl]-N-methylpyridinium; DAPI, 4,6-diamidino-2-phenylindol; LP1, Large extracellular loop 1; Mbp, Megabase pair; MNE, Mean normalized expression; MPP+, 1-methyl-4-phenylpyridinium; MS222, Tricaine methane sulfonate; MSF, Major Facilitator Superfamily; OAT, Organic anion transporter; OCT, Organic cation transporter; OCTN, Organic cation/carnitine transporter; ORCTL, Organic Cation Transporter-Like; SLC, solute carrier; TEA, Tetraethylammonium; TMD, transmembrane domain

## Additional files

Additional file 1: Figure S1. Extended phylogenetic tree of Slc22/slc22 protein family in vertebrates; Figure S2: Conserved synteny analysis of less characterized human and zebrafish Slc22/slc22 genes; Figure S3: Tissue expression profile of major zebrafish slc22 genes organized by individual genes; Table S1: Protein annotations with accession numbers of all analysed species; Table S2: Heatmap of zebrafish Slc22 gene tissue expression results. (XLSX 1109 kb) Additional file 2: Gene structure analysis of zebrafish and human slc22/Slc22. Structure of coding exons, exons and introns together with gene lengths. (XLSX 539 kb) Additional file 3: Transcription factor binding motif analysis of zebrafish and human slc22/Slc22 genes. Identification of potential sequence motifs and regulatory elements of human and zebrafish Slc22/slc22 genes. (XLSX 55 kb) Additional file 4: Amino acid sequence motif analysis of zebrafish and human Slc22/SLC22. Identification of characteristic Slc22 amino acid sequences with identified major facilitator superfamily (MFS) motif, amphiphilic solute facilitator (ASF) domain and OAT-specific motif. (XLSX 518 kb) Additional file 5: Analysis of zebrafish and human Slc22/SLC22 protein topologies. Secondary structures of Slc22/SLC22 proteins showing transmembrane domains and extracellular and intracellular loops. (XLSX 563 kb)
